# Hearing It Again and Again: On-Line Subcortical Plasticity in Humans

**DOI:** 10.1371/journal.pone.0013645

**Published:** 2010-10-26

**Authors:** Erika Skoe, Nina Kraus

**Affiliations:** 1 Auditory Neuroscience Laboratory, Northwestern University, Evanston, Illinois, United States of America; 2 Department of Communication Sciences and Disorders, Northwestern University, Evanston, Illinois, United States of America; 3 Department of Neurobiology and Physiology, Northwestern University, Evanston, Illinois, United States of America; 4 Department of Otolaryngology, Northwestern University, Chicago, Illinois, United States of America; University of Leuven, Belgium

## Abstract

**Background:**

Human brainstem activity is sensitive to local sound statistics, as reflected in an enhanced response in repetitive compared to pseudo-random stimulus conditions [1]. Here we probed the short-term time course of this enhancement using a paradigm that assessed how the local sound statistics (i.e., repetition *within* a five-note melody) interact with more global statistics (i.e., repetition *of* the melody).

**Methodology/Principal Findings:**

To test the hypothesis that subcortical repetition enhancement builds over time, we recorded auditory brainstem responses in young adults to a five-note melody containing a repeated note, and monitored how the response changed over the course of 1.5 hrs. By comparing response amplitudes over time, we found a robust time-dependent enhancement to the locally repeating note that was superimposed on a weaker enhancement of the globally repeating pattern.

**Conclusions/Significance:**

We provide the first demonstration of on-line subcortical plasticity in humans. This complements previous findings that experience-dependent subcortical plasticity can occur on a number of time scales, including life-long experiences with music and language, and short-term auditory training. Our results suggest that the incoming stimulus stream is constantly being monitored, even when the stimulus is physically invariant and attention is directed elsewhere, to augment the neural response to the most statistically salient features of the ongoing stimulus stream. These real-time transformations, which may subserve humans' strong disposition for grouping auditory objects, likely reflect a mix of local processes and corticofugal modulation arising from statistical regularities and the influences of expectation. Our results contribute to our understanding of the biological basis of statistical learning and initiate a new investigational approach relating to the time-course of subcortical plasticity. Although the reported time-dependent enhancements are believed to reflect universal neurophysiological processes, future experiments utilizing a larger array of stimuli are needed to establish the generalizability of our findings.

## Introduction

The ability to entrain to rhythmic, repetitive patterns is the cornerstone of a dynamic auditory system. Regularities are extracted from a sound sequence using local and global sound statistics, resulting in the development of expectancies for future sounds [2], [3], [4]. This “active search for regularity” is considered a universal process [5] that cuts across sensory modalities [6], [7] and is evident in human neonates [8], non-human primates [9] and rodents [10]. This implicit learning of patterns within novel sequences also occurs rapidly (within 2 minutes) and without training, reinforcement [11], [12], [13] or awareness [7].

Regularity detection mechanisms — including adaptation to statistically-probable stimuli and stream segregation — span the entire auditory pathway, extending to subcortical structures [14], [15], [16], [17], [18], [19]. In humans, the auditory brainstem response (ABR) offers a means to study subcortical regularity-detection mechanisms in a non-invasive manner. By recording ABRs to speech and music, subcortical enhancements have been observed in response to stimuli that are behaviorally relevant to the listener and have a high probability of occurrence. This experience-dependent modulation of the brainstem, which is thought to be under corticofugal control, occurs over the course of short-term (on the order of weeks) [20], [21] and lifelong auditory experience with behaviorally-relevant signals [1], [22], [23], [24], [25].

The brainstem's sensitivity to local sound statistics has recently been demonstrated in humans [1]. Chandrasekaran and colleagues found that the ABR to the speech syllable [da] elicits a larger sustained response when it is presented in a repetitive (i.e., predictable) context compared to when the same sound is presented pseudo-randomly within a set of seven other speech syllables. The degree of enhancement to repeating sounds was correlated with performance on a speech-in-noise task, suggesting that regularity-detection mechanisms might be involved when an auditory object must be separated from background noise.

The goal of the present study was to probe the short-term time course of the repetition effect observed in Chandrasekaran et al. (2009) using a paradigm that enabled us to observe how the local sound statistics (i.e., repetition *within* a five note melody) interact with more global statistics (i.e., repetition *of* the melody). If repetition-enhancement mechanisms are important for processing auditory scenes, the subcortical response should be continuously refined as the stimulus is repeated on both local and global time scales. To test the hypothesis that repetition enhancement builds over time, we recorded ABRs to a five-note melody containing a repeated note and monitored how the response to the locally and globally repeating elements changed over the course of the 1.5 hr recording. This analysis was preformed by dividing the experiment into blocks (each comprising the same number of stimulus representations) and comparing the response amplitudes across blocks. If the response did not change across blocks, this would indicate that ABRs to complex sounds are stable over prolonged repetitive stimulation. Such a result would be consistent with the literature showing that the ABRs to repeating simple stimuli are highly repeatable within- [26], [27] and across-sessions [21], [28], [29], [30], [31], [32], [33] for an individual subject. However, if the response to this complex stimulus does evolve, this would provide strong support for the argument that subcortical sensory systems are adaptive processors that adjust to the immediate sensory and behavioral context in a process involving both corticofugal and intrinsic circuits [1], [34], [35].

## Methods

All experimental protocols were reviewed and approved by Northwestern University's Institutional Review Board.

### Participants

Eleven volunteers (6 females; 19–30 years; M = 22.3 years) participated in this study. All participants had normal hearing as assessed by an audiometric screen (thresholds <25 dB HL for octave frequencies between 125–8000 Hz) [36] and normal click-evoked ABRs. Written informed consent was obtained from all participants.

### Stimulus

Auditory brainstem responses were recorded to a five-note piano melody (E3-E3-G#3-B3-E4, 1093 ms) that forms an ascending triad, a ubiquitous construct in Western music. The first and second notes were identical on all acoustic parameters.

**Figure 1:**
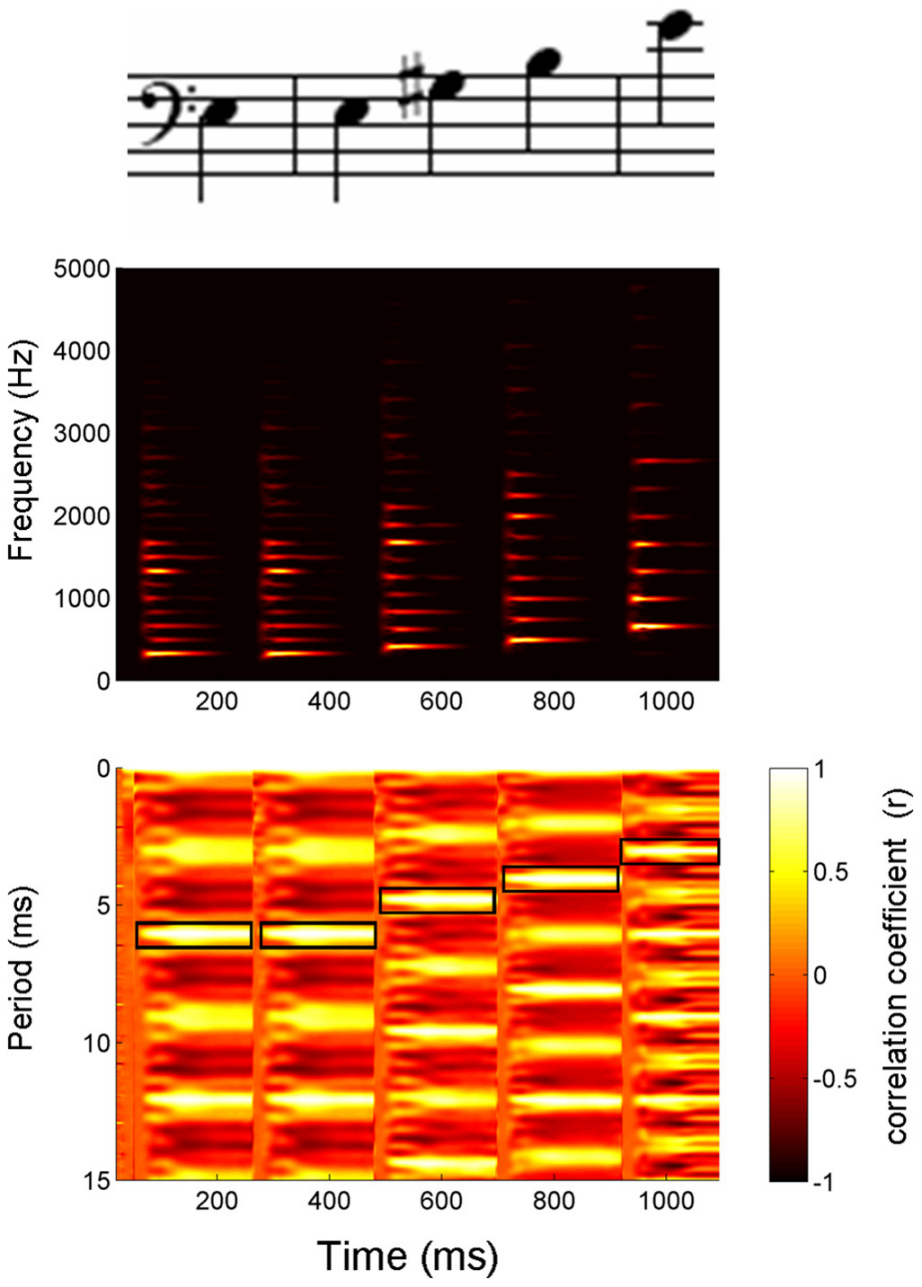
Description of the stimulus. (**Top**) The melody was composed of piano five notes, E3-E3-G#3-B3-E4. Notes 1 and 2 were acoustically identical. (**Middle**) Each ∼220 ms note had a rich harmonic structure that was dominated by the second harmonic (H2) (330, 330, 416, 494, 660 Hz, respectively), the lowest frequency in the spectrum of this “missing fundamental” stimulus. (**Bottom**) As shown in the stimulus autocorrelogram, the amplitudes of the harmonics interact to create a signal that is strongly modulated at the period of the fundamental frequency (F_0_), as evidenced by the brightest bands of color occurring at periods of 6.06, 6.06, 4.81, 4.05, 3.03 ms, respectively (marked by black boxes). The reciprocal of these periods correspond to 165, 165, 208, 247, 330 Hz, respectively. Following procedures described in Kraus and Skoe (2010) [45], the autocorrelogram was generated using a sliding-window cross-correlation function. The first time window encapsulated 0–40 ms of the stimulus, with each subsequent window starting 1 ms after the previous. Each 40-ms time window was cross-correlated with itself and degree of correlation at each time shift (y-axis) is plotted using a color scale, such that white represents the highest correlation. In this plot, the x-axis values refer to the center of each window (e.g., window 1 at 20 ms, window 2 at 21 ms, etc.) and the y-axis values refer to the time shift of the autocorrelation function.

**Figure 2:**
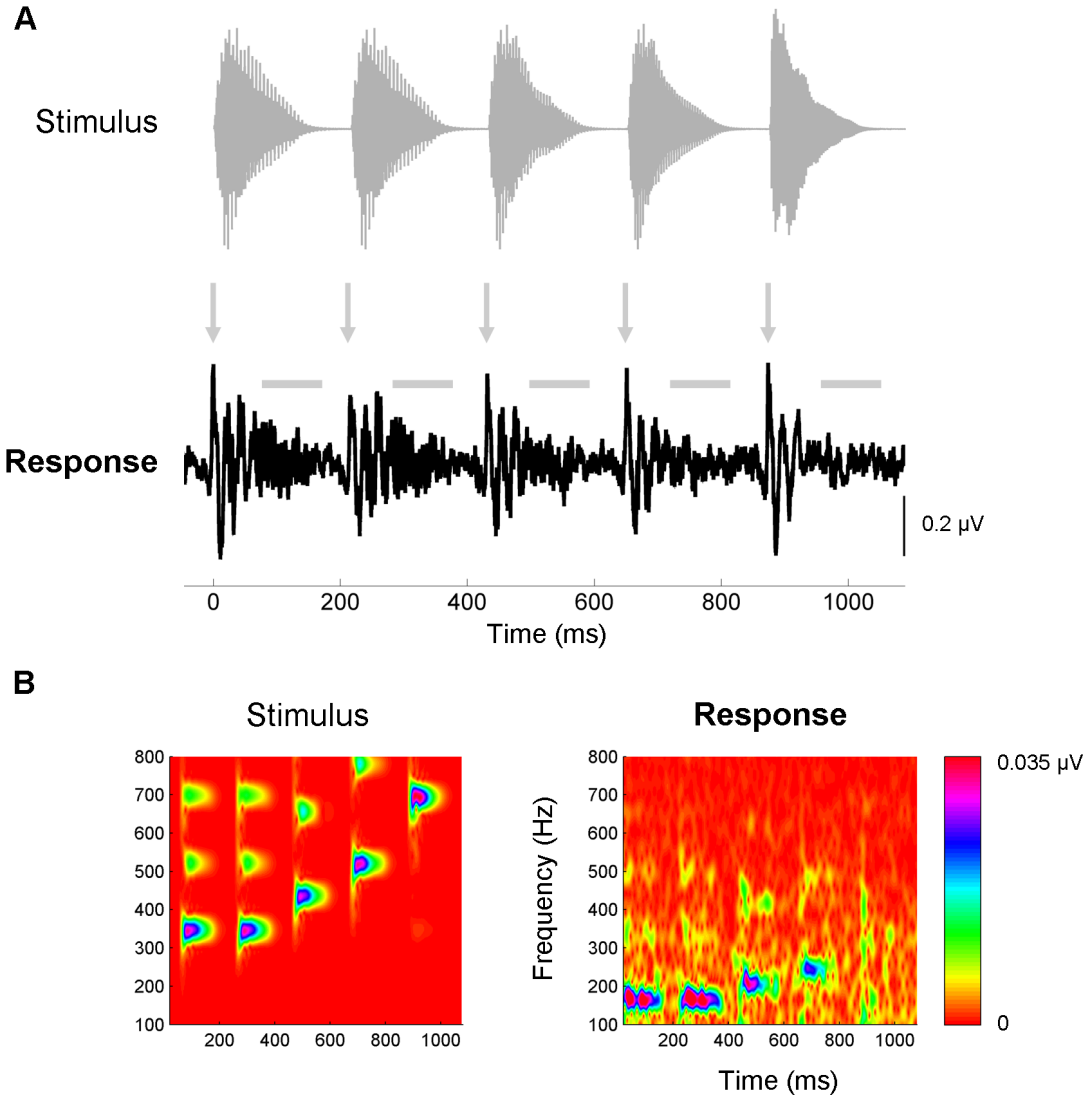
Description of the response. (**A**) Time domain. Percussive instruments, like the piano, have sharp attacks and rapid decays. As seen here, these aspects of the stimulus (top, gray) are preserved in the response (bottom, black). This is evidenced by large response peaks coinciding with the onset of each piano note (arrows). Horizontal bars identify the frequency-following response (FFR), the neural synchronization to the periodic aspects of each note. (**B**) Frequency domain. The stimulus (left) and response (right) spectrograms. Phase-locking to the fundamental (F_0_) and its second harmonic (H2) is observed in the FFR to each note. As predicted from the low-pass nature of brainstem phase-locking, the response to the F_0_ (165, 165, 208, 247, 330 Hz, respectively) is stronger than the response to resolved harmonics of the stimulus (H2 = 330, 330, 416, 494, 660 Hz, respectively). A representative subject is plotted.

Each harmonically complex note was created separately in Music Masterworks, a music composing software package (Aspire Software LLC, Golden, CO), using built-in piano timbres. All subsequent sound editing occurred in Adobe® Audition® 2.0 (Adobe Systems Incorporated, San Jose, CA). The final stimulus was formed by concatenating five individual sound files into a single 1093 ms WAV file. To prevent the introduction of a click when the individual notes combined into a single file, each note was trimmed at a zero crossing, after being time-compressed (while maintaining pitch). The final duration of each note was 216, 216, 220, 220, and 221 ms, respectively. Given the sharp amplitude decay that is characteristic of a percussive instrument like the piano, the notes in the melody were clearly separated in time (i.e., no silence was inserted between the notes) (Figures 1 and 2).

The data analyzed in this study were originally collected as part of a study examining brainstem encoding of virtual pitch. For this reason, the stimulus represented a “missing fundamental” sound, created by removing the fundamental frequency (F_0_) of each note (165, 165, 208, 247, and 330 Hz, respectively) through the application of a high-pass filter in Adobe® Audition®. As a result of this transform, the lowest and most prominent frequency of each note fell at the second harmonic of the F_0_ (330, 330, 416, 494, and 660 Hz, respectively) (Figure 1, middle). From a perceptual standpoint, the harmonics of a missing fundamental form a coherent auditory object that is perceived to be one octave lower [37], [38] (165, 165, 208, 247, and 330 Hz, respectively) than the lowest actual frequency. In the case of our five-note melody, although the F_0_s were spectrally absent, a frequency-following response (FFR) [39], [40] to the F_0_ of each note (Figure 2) was observed because the fundamental periodicities of the F_0_ s were present in the temporal envelope of the stimulus (Figure 1, bottom). This outcome is consistent with previous work utilizing virtual pitch stimuli [41], [42], [43], [44].

The stimulus was delivered by Gentask (Compumedics, Inc., Charlotte, NC) in alternating polarity at 80 dB SPL to the right ear through an ER-3A insert earphone (Etymotic Laboratories, Elk Grove Village, IL). See Skoe and Kraus 2010 [45] where methodological considerations of polarity are covered in depth. The five-note melody was played repeatedly for 1.5 hours with 64.4 ms of silence between repetitions; this interval of silence, which was kept short to minimize test time, is sufficient to elicit a perceptually distinct gap between each presentation of the melody.

### Procedure

The ABR, which is presumed to originate largely from the midbrain (inferior colliculus) [46], was collected at a sampling rate of 20 KHz (Neuroscan Acquire, Compumedics, Inc., Charlotte, NC) using a vertical electrode montage (Cz to ipsilateral earlobe, with the forehead serving as ground). Contact impedance was <5 kOhms for all Ag-AgCl electrodes.

During testing, subjects sat comfortably in a reclining chair in a sound attenuating room and viewed a movie of their choice. The movie soundtrack, which was set to < = 40 dB SPL, was audible to the left ear. This widely-employed passive collection technique enables the subject to remain awake yet motionless during testing [45], [47], [48].

Responses were processed off-line in Neuroscan Edit (Compumedics, Inc., Charlotte, NC) by filtering from 30–2000 Hz (12 dB/octave) and then epoching with a interval of −50 to 1100 ms (stimulus onset at 0 ms). The pre-stimulus period (−50 to 0 ms), during which there was no acoustic stimulation, served as a common noise floor baseline for all five notes. After baseline correcting to the mean voltage of the noise floor, trials with activity exceeding +/− 50 microvolts were considered artifacts and were excluded from the pool of available trials. After the artifact rejection process, there were ∼4000 remaining trials from which two sets of averages were created, each segmenting the recording into finer time intervals: (1) two sub-averages of ∼2000 trials, representing the first and second halves of the recording, respectively and (2) four sub-averages of ∼1000 trials, each representing one quarter of the test session (roughly 20 minutes of testing). Because ABRs do not emerge from the noise floor without averaging many hundred of trials together [33], [49] smaller timeframes could not be evaluated due to impoverished signal-to-noise ratios.

### Analysis

The ABR preserves many of the temporal and spectral characteristics of the evoking stimulus (Figure 2). As can been seen in Figure 2, the response to each note is characterized by two distinct response types [50], namely a transient onset response followed by a sustained FFR, reflecting the neural synchronization (phase-locking) to the periodic aspects of each note. Time (onset response) and frequency domain (FFR) measurements were made in MATLAB 7.0 (The Mathworks, Natnick NJ) and analyzed statistically in SPSS (Chicago, IL) after correcting for outliers.

The amplitude of the onset response was measured by calculating the average root-mean-square (RMS) amplitude over a 4-ms range surrounding the first peak of the onset complex. The center point of the RMS range for each ∼220 ms note (10, 225, 442, 661, and 884 ms, respectively) was chosen based on visual inspection of the grand-average response (across all subjects and trials). FFRs were visually identified to begin at 52, 267, 482, 700, and 930 ms (respectively) and extend for 100 ms. Note 5, which has the highest pitch and the greatest separation between successive harmonics (Figure 1, middle), did not elicit strong phase-locked activity (Figure 2), and was excluded from the FFR but not the onset analyses. The FFR was transformed to the spectral domain using the fast Fourier transform with zero padding. Zero padding is a common digital signal processing technique in which a string of zeros is appended onto the time domain waveform to increase the spectral estimates (in this case from 10 Hz to 1 Hz). The amplitudes of the response to the F_0_ and H2 were obtained for each subject for Notes 1–4 by finding the amplitude of the spectral peak nearest the frequency of the F_0_ and H2 (i.e., the nearest local maxima). Higher harmonic components were not reliably present in all subjects and were not measured. For the F_0_, the mean frequencies of the maxima for the four notes were 164.36, 164.00, 209.09, 245.00 Hz, respectively (SD = 2.87, 1.61, 5.75, 4.96 Hz, respectively). For H2 they were 328.45, 329.64, 414.91, 495.46 Hz, respectively (SD = 3.50, 5.35, 2.70, 4.13, respectively). Notes 1 and 2 did not differ statistically in terms of the frequency of the spectral peak that was analyzed.

Noise floor estimates were calculated by transforming the pre-stimulus period to the frequency domain. Then, on a note by note basis, the amplitude at the frequency corresponding to the FFR peak for each respective harmonic was found. For example, if for a particular subject, the H2 peak occurred at 329 Hz for Note 1, the noise floor of that peak was calculated as the amplitude at 329 Hz during the pre-stimulus period. Because the H2 peak may have occurred at a slightly different frequency for Notes 1 and 2, the noise floor estimates were not necessarily identical for the two notes for an individual subject.

## Results

### FFR-global repetition

With the exception of the F_0_ of Note 3, the mean amplitudes for F_0_ and H2 increased between the first and last halves of the recording. FFR peak amplitudes (means and standard deviations) are presented in Table 1 for F_0_ and H2.

**Table 1:** Mean amplitude of the fundamental frequency (F_0_) and second harmonic (H2) for each note for the first and last halves of the recording.

	Time Period (Half)	F_0_ Mean Amplitude (µV)	H2 Mean Amplitude (µV)
**Note 1**	First	0.041 (0.008)	0.016 (0.033)
	Last	0.046 (0.009)	0.020 (0.010)
**Note 2**	First	0.043 (0.010)	0.017 (0.007)
	Last	0.046 (0.012)	0.025 (0.006)
**Note 3**	First	0.025 (0.007)	0.015 (0.005)
	Last	0.023 (0.008)	0.017 (0.005)
**Note 4**	First	0.021 (0.005)	0.008 (0.004)
	Last	0.023 (0.012)	0.108 (0.005)

Standard deviations are reported in parentheses.

**Figure 3:**
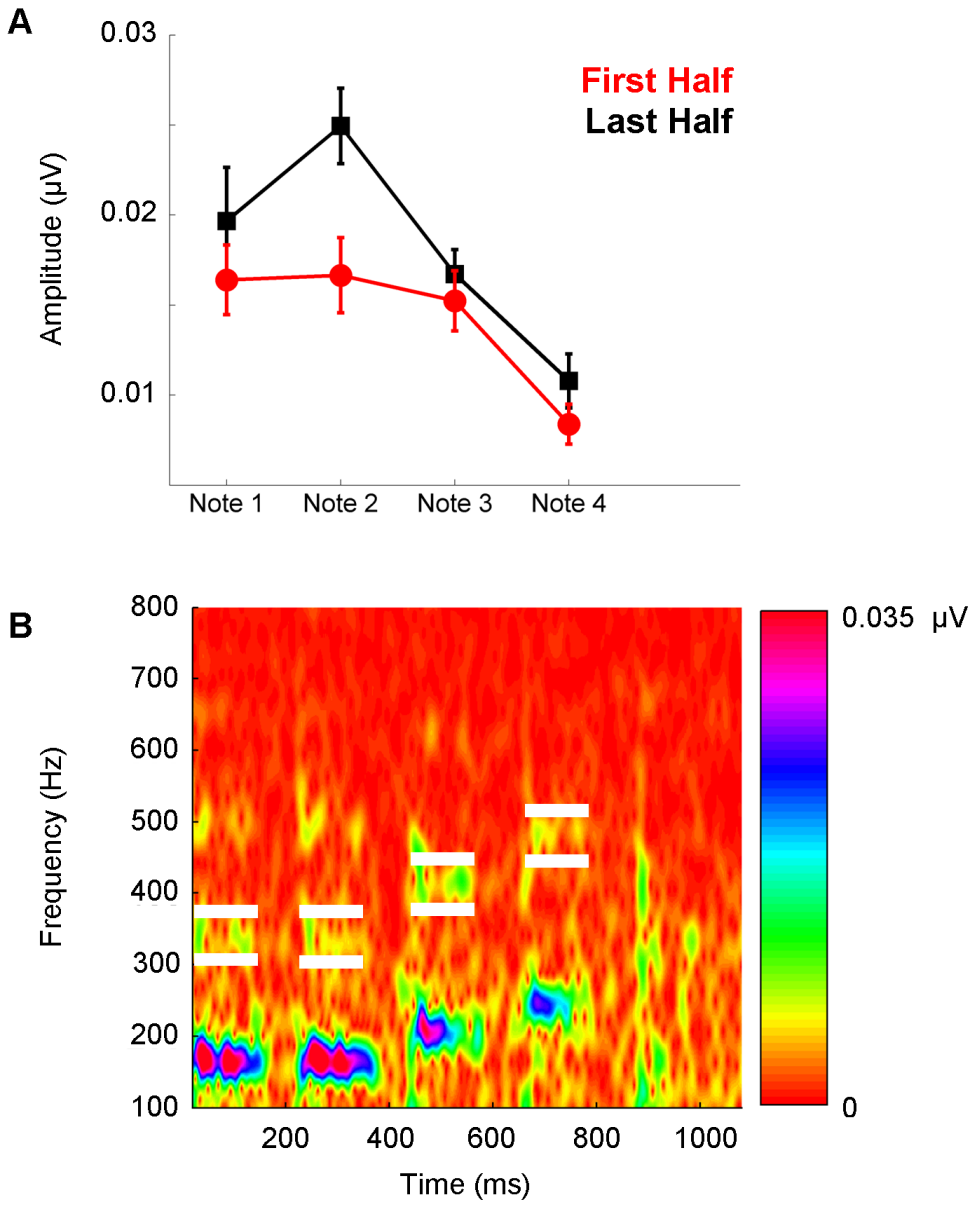
Repetition enhancement of the melody. (**A**) Across all notes, the frequency-following response to the second harmonic (H2) was larger during the second half of the recording session (red) compared to the first half (black). (**B**) White boxes bracket H2 for Notes 1–4 in the response spectrogram of a representative subject (averaged across all trials).

To determine statistically whether the FFR to the globally-repeating melody was enhanced through repetition, a 4×2 (Note × Time) repeated measures ANOVA (RMANOVA) was conducted separately for F_0_ and H2 (Note 5 was excluded, see Methods). Although a main effect of Note was found for F_0_ (F(3, 10)  = 15.158, p<0.0001), no main effect of Time (F(1, 10)  = 1.187, p = 0.301) nor an interaction was found (F(1, 10)  = 0.747, p = 0.532). In contrast, for H2, main effects of Note (F(3, 10)  = 15.158, p<0.00001) and Time (F(1, 10)  = 14.001, p = 0.004), in addition to a Note × Time interaction, were observed (F(1, 10)  = 4.231, p = 0.013) (Figure 3). Given the low-pass nature of brainstem phase-locking [39], [40] and the fact that Notes 3 and 4 are higher in pitch than Notes 1 and 2, the main effect of Note was expected for both H2 and F_0_.

The main effect of Time (Figure 3) for H2 reflects an increase in response amplitude for all notes between the first and second halves of the recording, with the average increase for the H2 of each note being 21.34%, 64.80%, 20.76% and 61.68%, respectively. Importantly, however, this increase in response amplitude did not reflect concomitant time-dependent changes in the noise floor (F(1, 10)  = 0.180, p = 0.680), even when noise floor estimates are extracted at 330 Hz, and not the corresponding peak frequency, for both Note 1 and Note 2 (F(1,10) = 0.804, p = 0.391). The different time effects for H2 and F_0_ could be an indication that the response to F_0_ is “at ceiling” within its dynamic range.

### FFR-local repetition

**Figure 4:**
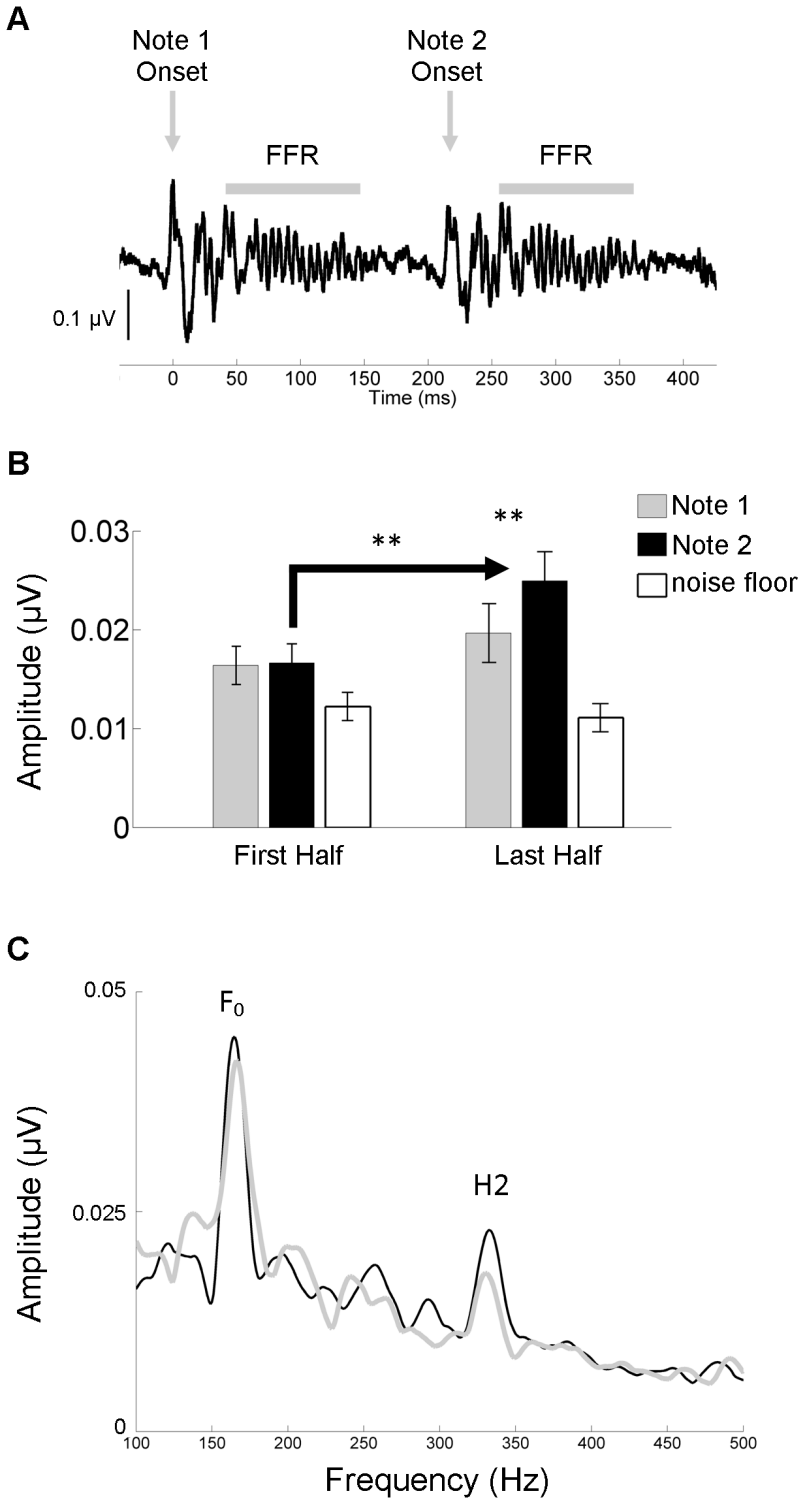
Local repetition enhancement over time. (**A**) The onset and frequency-following responses (FFRs) are plotted here in the time domain for Notes 1 and 2. In the stimulus, Notes 1 and 2 are identical in all respects. (**B**) The FFRs to Notes 1 and 2 did not differ in terms of the amplitude of second harmonic (H2) during the first half of the recording (left), but they did differ during the second half (right). While both Notes increased in amplitude over the recording session, the Note 2 enhancement was most pronounced (an average of 21.34% and 64.80% increase, respectively). This enhancement was not the result of increased activity in the noise floor (white bars represent the noise floor for Note 2 during the first and last halves). (**C**) The grand average spectrum for the last half of the recording is plotted for Notes 1 (gray) and 2 (black). The spectral peaks corresponding to the fundamental frequency (F_0_) and H2 are labeled.

**Figure 5:**
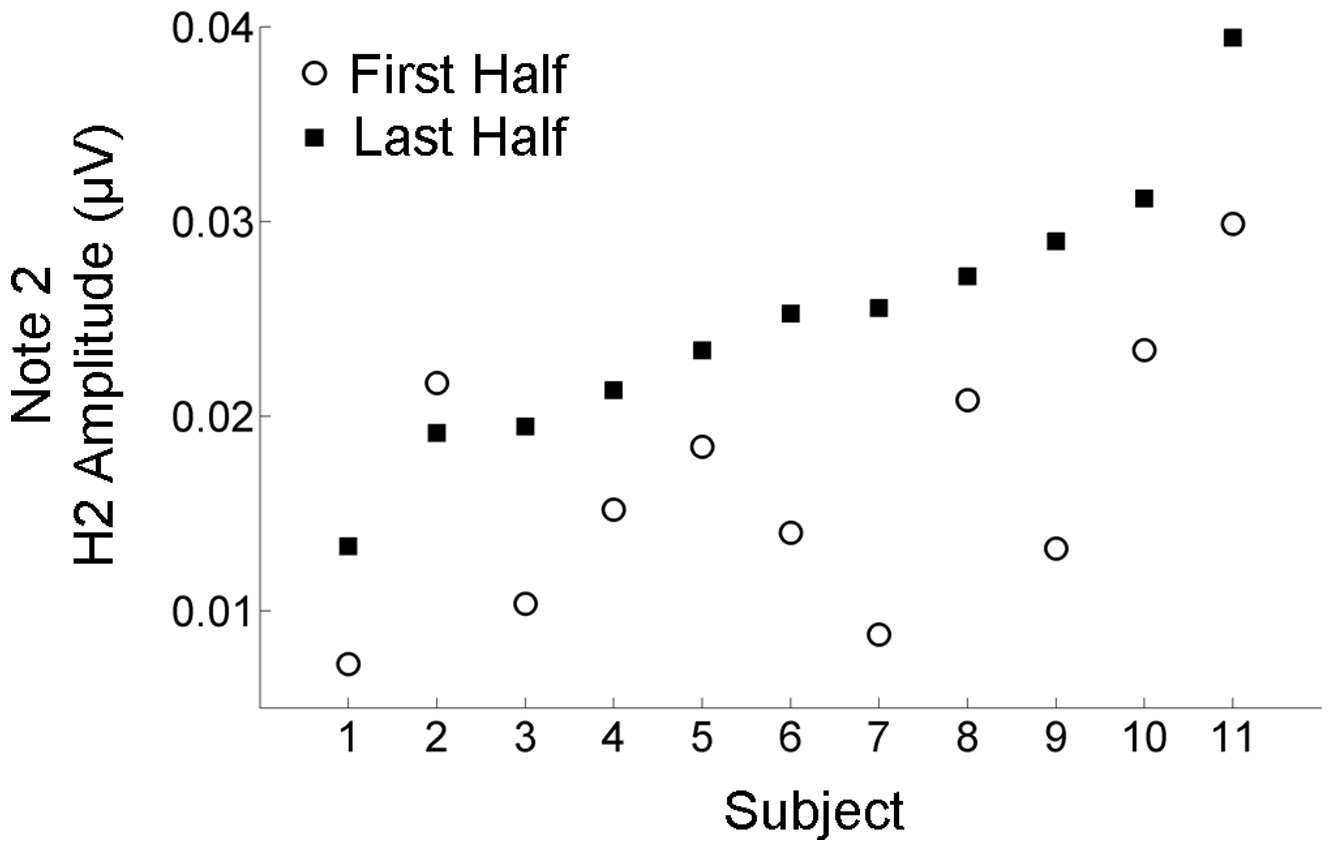
Time-dependent local enhancement of Note 2 in individual participants. For the frequency-following response to Note 2, the second harmonic (H2) amplitude is plotted for the first (open circles) and last (black squares) halves of the recording. The H2 enhancement, which ranged from 21.1–65.5%, was observed in 91% of the participants (10/11).

**Figure 6:**
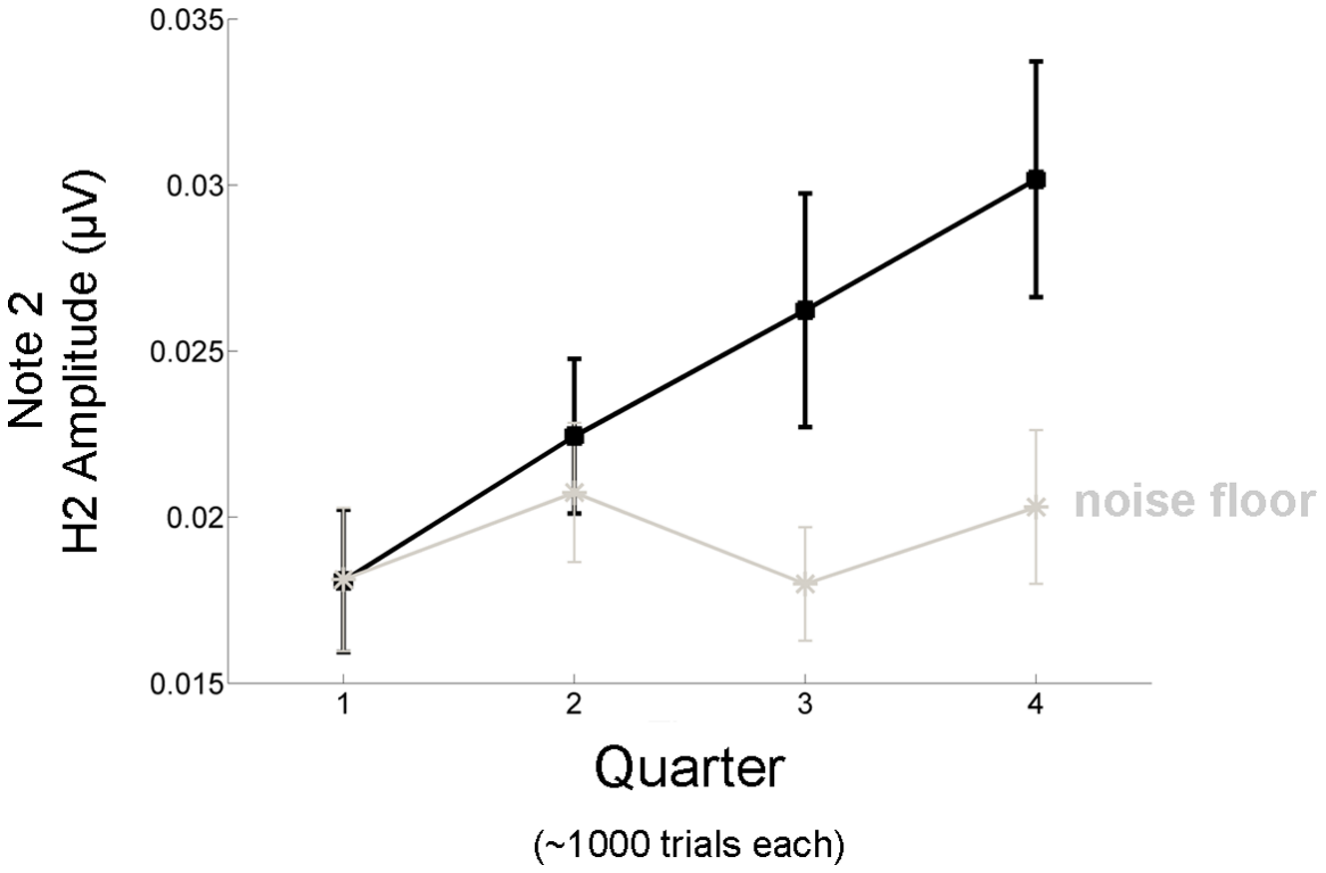
Local repetition enhancement of the frequency-following response (FFR) evolves throughout the test session. For Note 2 (black squares) the amplitude of the second harmonic (H2) increases monotonically over the test session. Each point represents the H2 amplitude derived from an average of ∼1000 trials. This increase in the FFR did not result from concomitant changes in the noise floor (gray stars).

To determine whether the H2 Note × Time interaction (reported above) was driven by the local repetition within the melody, post-hoc analyses (α = 0.0125) were performed that compared Notes 1 and 2. While the H2 amplitudes for Note 1 and Note 2 did not differ during the first half of the recording (t(10)  = −0.149, p = 0.885), they did differ during the second half (t(10)  = −3.689, p = 0.004) (Figure 4B). This Note 2 enhancement was highly reliable at the individual level (t(10)  = −5.180, p = 0.0004), with ten of the eleven subjects showing a clear local enhancement that ranged between 21.2–65.6% (Figure 5). The FFR amplitude results held when, instead of comparing the peak amplitudes, the frequency was fixed at 330 Hz for both Note 1 and Note 2 (first half: t(10)  = −0.191, p = 0.852; last half: t(10)  = −3.677, p = 0.004);

A final frequency-domain analysis evaluated how the H2 of Note 2 changed over smaller increments of time (four ∼20 minute blocks). For Note 2, a one-way ANOVA indicated a main effect of Time (F(1.1718, 17.179)  = 3.976, p = 0.043, p-value and degrees of freedom corrected for violations of sphericity). Based on the amplitude trajectory in Figure 6, H2 appears to be monotonically increasing. This effect was not driven by changes in the noise floor (F(3, 30)  = 0.596, p = 0.623). Moreover, the interaction that is observed in Figure 6 (F(3, 30)  = 2.844, p = 0.054) indicates that H2 emerges from the noise floor over the course of the recording.

### Onset-global repetition

**Figure 7:**
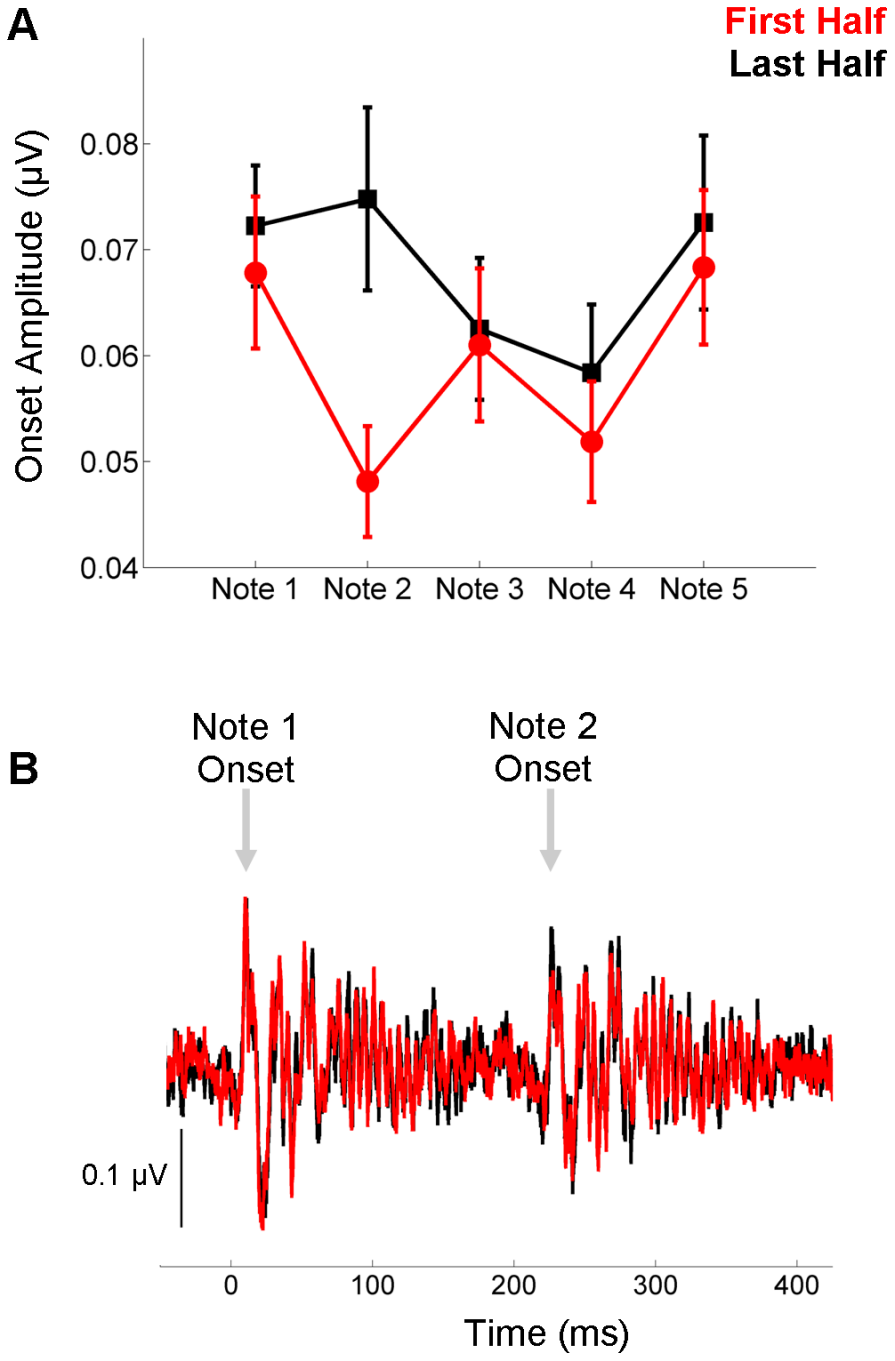
Repetition effects for the onset response. (**A**) For all notes, the onset response was larger during the second half of the recording session (red) compared to the first half (black). (**B**) As shown here in the time domain waveforms, the onset response to Note 2 is markedly bigger during the second half of the recording compared to the first.

The onset responses were analyzed by computing the RMS amplitude of the onset response peaks. Because the onset response is less temporally salient with only 1000 sweeps, this analysis focused only on how the response changed between the first and last halves of the recording. For all notes, the mean amplitude increased over the course of the recording (Table 2; Figure 7). This was confirmed statistically using a 5×2 RMANOVA (Note × Time) that included all notes. The results of the RMANOVA included a main effect of Time (F(1, 10)  = 8.165, p = 0.017) (Figure 7), which suggests that the response to the globally repeating pattern is accentuated over time. The main effect of Note and the Time × Note interaction were trending toward significance (F(4, 10)  = 2.327, p = 0.073, F(4, 10)  = 2.015, p = 0.111, respectively), likely reflective of the small sample size.

**Table 2:** Mean onset amplitude for each note for the first and last halves of the recording.

	Time Period (Half)	Mean Amplitude (µV)
**Note 1**	First	0.069 (0.023)
	Last	0.073 (0.021)
**Note 2**	First	0.048 (0.017)
	Last	0.074 (0.030)
**Note 3**	First	0.061 (0.024)
	Last	0.063 (0.022)
**Note 4**	First	0.052 (0.018)
	Last	0.058 (0.021)
**Note 5**	First	0.068 (0.024)
	Last	0.073 (0.027)

Standard deviations are reported in parenthesis.

### Onset-local repetition

Because the Time × Note interaction failed to be statistically significant, post-hoc analyses comparing the onset amplitudes for Note 1 to Note 2 are not valid. However, consistent with FFR results, the onset amplitude of Note 2 increased on average by 28%, which represents more than a 500% increase over Note 1 or the other notes (4.72%, −0.04%, 1.33% and 4.20% for Notes 1, 3, 4 and 5, respectively).

### Summary

Taken together, the FFR and Onset results suggest that the enhancement to the locally-repeating note was superimposed on a weaker enhancement to the globally-repeating motif.

## Discussion

We provide the first demonstration that human subcortical activity evolves in response to both the global and local statistical regularities within the ongoing stimulus stream. In this case, the global regularity refers to the repetition of the entire melody and the local regularity refers to the repetition of a note within the melody. In addition to showing that the subcortical representation of the melody became stronger over time, we found a robust enhancement to the repeated note (Note 2) that appears to develop monotonically over the 1.5-hour session. Although Notes 1 and 2 are acoustically indistinguishable, their positions within the melody confer different local statistics, despite having identical global statistics (i.e., both occur 4000 times during the recording). Note 2′s statistical role is reinforced by it being the repetition of the preceding note. Thus, the enhancement of Note 2, relative to Note 1 that develops over time may result from the influences of a locally repeating sound being repeated on a global scale. This robust enhancement could reflect of a schema-driven grouping strategy (i.e., grouping based on familiar patterns) [51] that results in two physically identical sounds eliciting non-identical responses as the melody is repeated continuously.

By showing that the second harmonic of Note 2 emerges from the noise floor with repetition, our findings reinforce the notion that the subcortical representation of complex sound is shaped by its immediate acoustic context to improve signal quality and ‘tag’ relevant features of the signal [1], [52]. Thus, it appears that the brainstem, likely as a consequence of the statistical enhancement of intrinsic circuitry and corticofugal influence, locks onto temporal patterns occurring on multiple time scales [53], such as a local repetition within a recurring melody. These processes may act in concert with the listener's musical knowledge and expectations to emphasize the perceptually salient features within a continuous stream.

### Repetition enhancement

Our results replicate and extend previous work in humans showing that passively-elicited subcortical [1] and cortical responses [47], [48], [54] are enhanced when a single sound is repeated. Thus, the repetition enhancement effect first demonstrated at a subcortical level by Chandrasekaran et al. (2009) seems to generalizes to repetition occurring in a number of different forms, such as single sound played repeatedly (i.e., the ‘da’ sound in [1]), a repeating melody, and a repeating note within a melody. Consequently, we view this effect as a general phenomenon that should apply to repetitive patterned sequences composed of simpler units (i.e., pure tones) as well as sequences in which the repeated note is embedded inside the stimulus (e.g., G#3-E3-B3-B3-C3). However, based on differences that were observed among the notes of the melody, we predict that the extent and time-dependent trajectory of the enhancement may not be equivalent across stimuli. Instead, the pattern of the on-line plasticity is likely dictated by the complexity of the stimulus, as well as the statistical features of the repetition.

While others have reported with-in and across-session enhancements of cortical potentials to repeated stimulation [47], [54], this is the first to show that the amplitude of the auditory brainstem response changes in a systematic fashion over the course of a single session. Within-session variability is typically found to be quite low for the traditional click-evoked ABR [26], [27], which given the brief nature of the stimulus (1 microsecond) and the rapid rate of presentation (>10/s), can be elicited in comparatively abbreviated recording sessions. A similar level of stability has been found in the limited reports of speech-ABR inter-session comparisons [30], [55], which again were based on responses to comparatively short (40 ms) and rapidly presented stimuli. Thus, the novelty of our findings may be the consequence of using a 1.1 second complex stimulus sequence and recording over an extended time period. Another explanation for why such time-dependent enhancements have not been observed previously for traditional click-ABRs is that our analyses focused on the amplitude of the onset and FFR waves, two metrics not typically used in a clinical setting. This is because amplitudes, unlike temporal measurements, tend to be highly variable even in the normal population [33]. Moreover, given that (to the best of our knowledge) there are no other reports in the literature that have used frequency domain measurements to examine the intra-session stability of the FFR to pure tones or more complex sounds, we cannot fully judge the novelty of our results until further investigations have been made. Without further research, it is not known whether the time-dependent buildup of the FFR occurs only for complex stimulus sequences or whether it would be evident for any repeated stimulus. To probe this further, future studies should employ a variety of other stimulus conditions and recording paradigms, including simpler acoustic units, longer tone sequences, unfamiliar melodic constructions, continuous streams (no silence between stimuli), sequences in which the repeated notes are not adjacent (e.g., E3-G#3-E3-B3-C3), passive and active listening paradigms, and well as shorter and repeated test sessions [47].

### Neural mechanisms and time course

Stimulus specific adaptation, representing a reduction in neural activity in response to repeated stimulation, is a well established effect. This phenomenon, which is evident in single neurons at cortical and subcortical levels, occurs very rapidly (i.e., within seconds) [14], [15], [17], [19], [53], [56], lasts until a novel stimulus is encountered, and as argued by Malmierca and colleagues [15], is assumed to be generated by local circuitry. The mechanisms that underlie the subcortical repetition enhancement of our complex stimulus are likely altogether different from those associated with neural adaptation. Instead we propose that that the observed pattern of subcortical on-line plasticity results from the statistical enhancement of intrinsic circuitry interacting with top-down influences such as auditory memory, musical knowledge, expectation and/or grouping via the corticofugal pathway. This is argument is consistent with that made by Tremblay and colleagues to explain the different impacts of stimulus repetition on the N1 and P2 components of the P1-N1-P2 complex [47]. They argue that the rapid and robust (within session) attenuation of N1 arises from bottom-up processes that overtime influence top-down (i.e., cortico-cortico) connections linked to auditory memory to produce neural enhancements of P2 to repetitive stimulation.

Although our recording paradigm does not permit individual trials to be evaluated, the apparent monotonic increase suggests that the repetition enhancement is initiated early in the recording session and grows with each successive trial. The slow-time course of the observed enhancement also points to corticofugal involvement. Effects of corticofugal modulation are known to occur within a few minutes of the onset of cortical activation, then build continuously until cortical activation is ceased, after which a slow recovery is observed (up to 3 hours) [52], [57], [58]. Consistent with our results, corticofugal modulation can be multi-parametric, operating along multiple acoustical domains (time, frequency and amplitude) to improve the input to the cortex (reviewed in [34]). This egocentric selection by the cortex, which emphasizes behaviorally-relevant and frequently occurring signals, results in increased response amplitudes, sharper neural tuning and decreased response latency for subcortical neurons that are matched to the parameters of the characterizing sound [52], [57], [58]. In animal models, this corticofugal modulation can result when an auditory stimulus is paired with cortical stimulation [52], [57], [59] or a conditioned stimulus (e.g., leg shock) [58], but also when a sound is played repeatedly in an unpaired condition [57], [60]. Because our subjects were not actively engaged in a behavioral task, the build-up over time is assumed to reflect the continuous adjustment of subcortical function by the cortex that arises from the experience of listening to repetitive stimulation [60], a viewpoint consistent with that of Yan and Suga [60].

### Future directions

Our findings pave the way for a new investigational approach for studying the time course of subcortical plasticity and the potential role that the corticofugal pathway plays in auditory learning in humans [61]. By utilizing more complex stimulus statistics that approximate those found in language [11], [12], this experimental paradigm could provide a real-time window into subcortical function *during the learning process itself* [62], [63]. This future work, which may help to reveal the neural underpinnings of learning impairments [64], [65] and expertise, is supported by mounting evidence that ABRs provide neural signatures of auditory processing in expert (e.g., musicians) and non-expert learners (e.g., dyslexic children) [23], [25].

### Implications and summary

In combination with single-cell recordings, our results suggest that subcortical neurons have dynamic properties covering multiple timescales, from milliseconds to hours. By locking onto rapid changes and local and global patterns within an auditory scene, listeners can egocentrically adjust to the statistics of many ecologically-diverse environments to respond maximally to behaviorally-relevant signals such as speech and music that occur over many different time scales [53]. In this case, the extended repetition of the melody may also invoke a feedback loop in which the auditory system operates in an oscillatory mode, reinforcing the rhythmic nature of the passively-attended stimulus [66], [67]. Thus, both exogenous and endogenous factors may facilitate the enhancement of the perceptually-relevant features of the signal. These real-time subcortical transformations, which may subserve humans' strong predisposition for grouping, likely reflect a mix of local and top-down processes that are influenced by implicit and explicit knowledge about the auditory stimulus and expectation [68]. As argued by Winkler and colleagues (2009) [4], predictable patterns can be extracted from the on-going stimulus stream without focused attention, which may account for the effects observed in cortical potentials recorded from comatose and nonconscious patients [69], [70] newborns [71], as well as the present results collected under passive listening conditions.
